# Emerging Techniques in Minimally Invasive Surgery in Hematologic Malignancies

**DOI:** 10.1111/ejh.70052

**Published:** 2025-10-24

**Authors:** Alejandro Chara, Katlyn G. McKay, Harold N. Lovvorn, Joseph C. Fusco

**Affiliations:** ^1^ Section of Surgical Sciences Vanderbilt University Medical Center Nashville Tennessee USA; ^2^ Department of Pediatric Surgery Vanderbilt University Medical Center Nashville Tennessee USA

**Keywords:** augmented reality, laparoscopy, minimally invasive surgery, robotic surgery, thoracoscopy

## Abstract

Hematologic malignancies represent the most common cancers in children. While the mainstays of treatment are chemotherapy and potentially hematopoietic stem cell transplant, minimally invasive surgery (MIS) has a role in the diagnosis and management of complications related both to disease and therapy as well as common pediatric surgical conditions. MIS can be considered in the management of adverse events of disease and therapy such as pneumatosis, bowel perforation, or pneumoperitoneum in stable patients as a first‐look alternative to open surgery. Additionally, given the extent of therapy for many children, common surgical problems such as the need for enteral access, appendicitis, and cholecystitis can occur, and laparoscopic options should be considered for management to enable a faster return to chemotherapy. Finally, MIS can assist with diagnosis through lymph node or tissue biopsy. The utilization of MIS in pediatric patients with hematologic malignancies is a safe and feasible approach for a variety of surgical problems. Nevertheless, current evidence is largely limited to retrospective series and case reports, and prospective multicenter studies will be required to validate these approaches and emerging technologies such as augmented reality and robotics.

## 1 Introduction

Hematologic malignancies are among the most common cancers in children, with leukemia and lymphoma accounting for 28% and 19% of all pediatric cancers, respectively [1]. Fortunately, the overall survival of pediatric hematologic malignancies has reached 80%–90% over the recent decades [2]. These dramatic improvements are mainly due to advances in chemotherapies that target cellular signaling pathways, novel immunotherapies, and checkpoint inhibitors. For example, acute lymphoblastic leukemia (ALL), the most common pediatric cancer, has an overall 5‐year survival of over 90%, largely due to the use of chemotherapy, tyrosine kinase inhibitors, monoclonal antibodies, and highly specialized chimeric antigen receptor (CAR) T‐cells in patients refractory to chemotherapy [3]. Likewise, monoclonal antibodies, such as Rituximab for non‐Hodgkin lymphoma, immunotherapy, and targeted therapies have improved the survival in children with lymphoma [4, 5]. Stem cell transplantation is another option, especially for refractory disease [2]. Surgery is limited in the treatment of hematologic malignancies. However, it does play a critical role in certain instances, including diagnosis and management of gastrointestinal or intrathoracic manifestations, intraabdominal complications from therapy, development of common surgical conditions in patients undergoing therapy, and staging via excisional lymph node biopsies [6].

Minimally invasive surgery (MIS) for oncologic patients was initially introduced in 1995 by Holcomb et al., demonstrating in a single‐site retrospective study that laparoscopic or thoracoscopic biopsies or assessments for staging purposes were highly accurate and carried minimal morbidity. Of the 85 patients included, 16 of these patients had lymphoma or leukemia. The indications for operating in these patients were primarily for tissue diagnosis of their primary tumor or assessment of infectious complications, including liver abscesses [7]. Since this time, the role of MIS has developed into a safe and effective operative approach for complete resection of intracavitary solid tumors [8, 9]. The presumed benefits of minimally invasive approaches include decreases in postoperative pain, shorter hospital stays, and less morbidity. Prior literature has demonstrated that MIS is feasible for resection of pediatric embryonal tumors without compromising oncologic outcomes, including local recurrence or relapse rates [10]. Similarly, pediatric patients with neoadjuvant‐treated Wilms tumors resected in an MIS fashion had a significantly decreased time to resume chemotherapy compared to an open approach [11]. While MIS approaches to solid‐organ malignancies are becoming more established and data‐driven, fewer data regarding the utility of MIS in the diagnosis and management of hematologic malignancies have been published. This review explores the use of MIS in these scenarios.

## 2 Bowel Perforation

Although rare, bowel perforation is one of the most morbid gastrointestinal complications in pediatric patients with hematologic malignancies [12]. Perforations typically present during the second half of the induction period of chemotherapy and occur in the distal small bowel and colon [13, 14]. Patients endorse a range of abdominal symptoms, including pain, distension, nausea, vomiting, diarrhea, and peritonitis [13, 14]. Diagnosis is typically found with cross‐sectional imaging, although some cases can be diagnosed with pneumoperitoneum on an abdominal X‐ray. Bowel perforation can result from tumor invasion, rapid tumor lysis with chemotherapy, or tissue necrosis. Bowel perforation, especially duodenal perforation, can rarely be the presenting sign of intestinal lymphoma. Therefore, it is crucial for pediatric surgeons to have a high index of suspicion when otherwise healthy children have an unexpected bowel perforation. In these scenarios, ulcer biopsy and thorough evaluation for peritoneal disease are paramount adjuncts [15]. Interestingly, perforation during chemotherapy is associated with better outcomes with continuation of treatment regimens, whereas perforation before chemotherapy is associated with delays in the start of treatment and higher morbidity [13, 15].

Perforations due to tumor burden or neutropenic enterocolitis, also known as typhlitis, are among the more common reasons for emergency surgery in pediatric patients with hematologic malignancies. One retrospective study of 240 pediatric patients with primary intestinal lymphoma found a perforation rate of 6.7%. Burkitt's lymphoma was the predominant lymphoma type at 92.5%, and patients had a median age of 5.3 years [13]. The current standard practice for treatment in bowel perforation is exploratory laparotomy with resection of the affected portion and anastomosis versus ostomy creation. Data on the use of laparoscopy in this scenario are lacking, with no large prospective studies available. Cases of laparoscopic repair of bowel perforation have been documented in non‐traumatic injuries but rarely in patients with hematologic malignancies. In these cases, laparoscopy was used in hemodynamically stable patients with localized perforation on CT imaging or as a means to identify the site of perforation prior to converting to open surgery [14, 16, 17].

**FIGURE 1:**
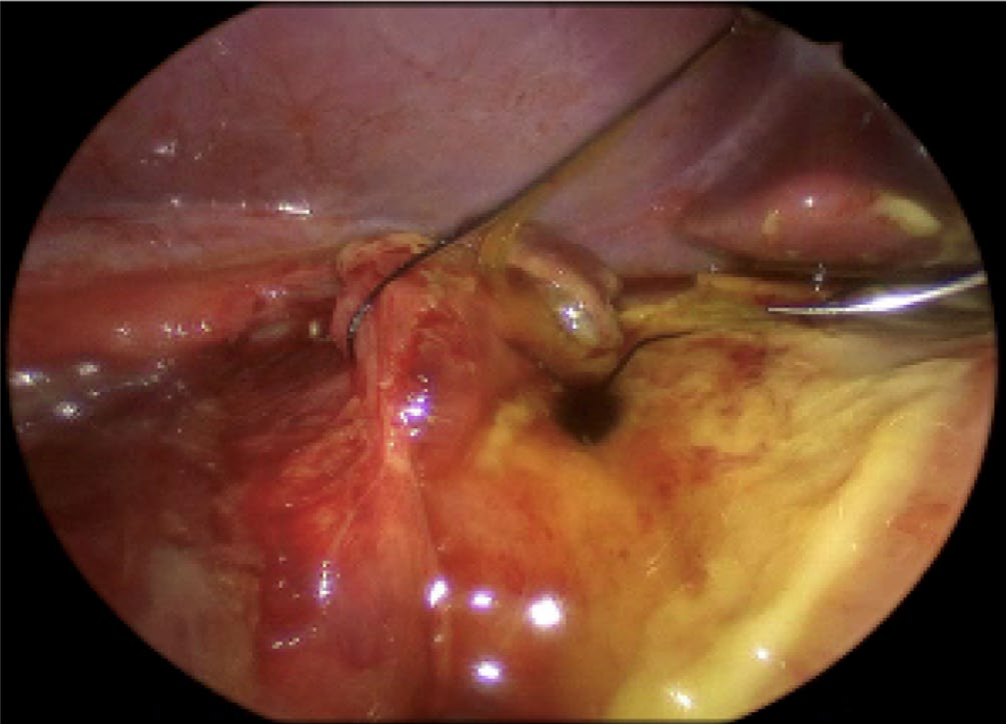
Laparoscopic repair of a duodenal perforation in a child with acute lymphoblastic leukemia. This example illustrates the feasibility of minimally invasive techniques in managing gastrointestinal complications of chemotherapy, avoiding the morbidity of open laparotomy.

Similarly, laparoscopy has not been thoroughly studied in typhlitis. Typhlitis typically is caused by the cytotoxic effects of chemotherapy leading to mucosal injury resulting in an acute, life‐threatening inflammatory process that most commonly occurs in the cecum [18]. Management is generally supportive, with broad‐spectrum antibiotics, bowel rest, and pain management. Surgery is reserved for cases of bowel perforation (Figure 1) or clinical decline despite aggressive medical management [19]. Laparoscopy has been used as a diagnostic tool when there is ambiguity in the etiology, but rarely is it used as a therapeutic approach. Some data suggest that laparoscopy could be beneficial in hemodynamically stable patients, but further studies are needed.

## 3 Intussusception and Bowel Obstruction

Intussusception is a common cause of acute abdominal pain in children, occurring when a segment of intestine telescopes into an adjacent segment of bowel at a lead point. There are different types, with the most common being ileocolic intussusception, in which the small bowel telescopes into the large bowel. Intussusception can be diagnosed via ultrasound or computed tomography scan, where imaging shows the intussuscepted bowel as well as potentially signs of pathologic lead points, such as thickened bowel wall or enlarged lymph nodes [20]. With regard to pediatric hematologic diseases, the most common cause of intussusception is gastrointestinal lymphoma, particularly B‐cell non‐Hodgkin lymphoma [20]. Gastrointestinal lymphomas account for 6.5% of pathologic lead points [21].

If no evidence of peritonitis or shock is present, the first line of treatment is generally pneumatic reduction of the intussusception. However, reduction is often not successful when the lead point is pathologic, such as a lymphoma. The rate of successful reduction with air enema drops significantly from ~92% in patients without lymphoma to ~40% in patients with lymphoma [20]. Surgical reduction via laparotomy or laparoscopy is an option when the intussusception appears pathologic on imaging or when air enema reduction is unsuccessful. Laparoscopic reduction has been shown to be an adequate management for intussusception, with success rates between 79%–90% and comparable outcomes with shorter hospital stays, but can be challenging with bulky, lymphoma‐involved mesenteric lymph nodes [22, 23, 24, 25]. The conversion rate to an open approach ranges from 5% to 12%, making laparoscopy a suitable initial approach [23, 24].

Bowel obstruction is another highly morbid complication of gastrointestinal hematologic malignancies, especially lymphomas. Several etiologies of bowel obstruction occur in these patients: sequela of intussusception, extrinsic compression by tumor on the bowel, or tumor involvement within the bowel itself. Bowel obstruction manifests in up to 17% of pediatric gastrointestinal lymphoma cases and most commonly occurs in the ileocecal region [20, 26, 27]. Supportive care and medical management of bowel obstructions are first‐line approaches in the absence of peritonitis or closed‐loop appearance on imaging, as these nonoperative strategies allow for more expedited treatment with systemic chemotherapy, which often rapidly improves the obstruction [26, 27, 28]. Surgery is reserved for cases where medical management has failed or if perforation is present [29, 30]. Emergency laparotomy is the most common approach to relieve bowel obstruction, but no large‐scale data evaluating the use of MIS in these settings exist. However, several case reports and small case series suggest laparoscopy offers favorable outcomes with improved morbidity in select patient populations [29, 30, 31].

## 4 Pneumoperitoneum After Stem Cell Transplant

The treatment algorithm for a variety of pediatric hematologic malignancies, including leukemia and lymphoma subtypes, often includes hematopoietic stem cell transplantation (HSCT) to achieve disease eradication or in children with relapsed and refractory disease [32, 33]. A common complication requiring evaluation from pediatric surgeons after HSCT is the development of pneumatosis intestinalis (PI) or even pneumoperitoneum. The cause of pneumatosis in these patients is still unclear; one suggested mechanism proposes that the immunosuppression required in HSCT leads to regression of lymphoid tissues and a weakening of the mucosal structural integrity. Alternatively, graft versus host disease has been postulated as a mechanism to incite PI through disruption of microbial diversity. Together, these changes may render the bowel wall susceptible to dissection by intraluminal gas, leading to pneumatosis and ultimately pneumoperitoneum in some cases [34].

The question regarding medical versus surgical management of pneumatosis remains outstanding. In a single‐site, retrospective study of patients who underwent HSCT, 10.1% developed PI, with 44.4% of these patients progressing to pneumoperitoneum. Of these patients, graft versus host disease was the major cause of mortality [35]. In this study, all patients were managed without surgical intervention despite the presence of “benign” pneumoperitoneum in several patients. Similarly, a case report exists of a child who developed graft versus host disease with massive intestinal pneumatosis and pneumoperitoneum and was managed conservatively with bowel rest as he remained asymptomatic, suggesting this may be a reasonable treatment strategy [36]. A single‐site study of 53 patients with PI after HSCT requested surgical consultation based on the presence of symptoms in just 13% of patients, and no interventions were required [37]. No deaths were attributed to pneumatosis, and one autopsy demonstrated no perforation despite the presence of pneumoperitoneum on imaging. The current literature suggests that surgical intervention is likely not required in asymptomatic HSCT patients with PI or pneumoperitoneum on imaging. However, one role for MIS could be a diagnostic laparoscopy in patients with pneumoperitoneum with an acute abdomen. Given the immunocompromised nature of these children, minimizing the extent of surgical intervention is fundamental, and starting with a diagnostic laparoscopy could be beneficial [38].

## 5 Common Pediatric Surgery Issues in Hematologic Malignancies

MIS approaches are also important to consider when managing common surgical issues in children with hematologic malignancies. Chemotherapy for these children can last several years and can contribute to surgical pathologies due to its immunosuppressive effects. Emergent surgical pathologies such as appendicitis and cholecystitis, as well as routine interventions like gastrostomy tube (G‐tube) placement, carry a higher risk in these patients. It is critical for these patients to return to treatment expeditiously; therefore, a minimally invasive approach is preferred when possible.

Laparoscopic appendectomy is one of the most common abdominal surgeries in children. The rate of acute appendicitis in pediatric hematologic malignancies is less than 1% [39]. Laparoscopic appendectomy has been shown to be safe and effective while also reducing the delay in returning to chemotherapy compared to open surgery or conservative antibiotic management [40, 41, 42]. Some centers opt to delay appendectomy and instead manage with antibiotics while awaiting partial hematologic response. However, this strategy is associated with higher rates of delayed surgery, longer hospital stays, and increased risks of appendiceal perforation [42, 43, 44]. The benefits of upfront laparoscopic appendectomy have also been shown in patients with severe neutropenia after undergoing adequate perioperative transfusions [43].

Acalculous cholecystitis is another surgical pathology that affects immunosuppressed patients. The incidence of acalculous cholecystitis in pediatric hematologic malignancies is estimated to be between 1% and 2% [45, 46]. Patients undergoing intensive chemotherapy or stem cell transplant are particularly prone to acalculous cholecystitis. Their immunosuppressed state increases the risk of bile stasis, ischemia, infection, and direct mucosal injury from chemotherapy [45, 47, 48]. Management is primarily with supportive care, antibiotics, and percutaneous cholecystostomy [46, 49]. Surgery is usually a last resort or done in refractory cases as most patients who develop acalculous cholecystitis are critically ill, making them poor surgical candidates [50]. When surgery is indicated, such as in the case of failure of medical management, gallbladder gangrene, or perforation, laparoscopic cholecystectomy is the preferred approach due to its less‐invasive modality and lower morbidity on patients who are already critically ill [51, 52, 53].

Chemotherapy and stem cell transplantation significantly hinder the nutritional status of pediatric patients. Thus, many patients require supplemental nutrition via tube feeds. One common option is nasogastric tube feeds. However, gastrostomy tubes are usually more comfortable for patients when nutritional support is required long‐term [54, 55]. G‐tubes can be placed endoscopically (percutaneous endoscopic gastrostomy (PEG) tubes), laparoscopically, or open. PEG and laparoscopic G‐tubes are the most common approaches performed given their minimally invasive technique. Multiple meta‐analyses have demonstrated that laparoscopic G‐tube placement carries fewer major complications, including the need for emergency surgery for leaks, obstruction, or perforation, than PEG tubes in children [56]. The direct visualization of the stomach during laparoscopic G‐tubes is especially beneficial in patients with prior abdominal surgeries, a lack of an adequate window to the stomach, or severe malnutrition [57].

Splenectomy in pediatric hematologic malignancies is reserved for indications such as symptomatic hypersplenism, refractory cytopenias due to splenic sequestration, or splenic ruptures [58]. This procedure is more commonly performed in patients with acute or chronic leukemias and can be used to improve anemia, thrombocytopenia, and reduce transfusion requirements [59]. The decision to proceed with splenectomy is made on an individual basis and usually involves a multidisciplinary approach. Laparoscopic splenectomy is the favored technique in these cases. It results in shorter hospital stays, reduced postoperative pain, and a higher safety profile compared to open splenectomies [59, 60, 61].

**FIGURE 2:**
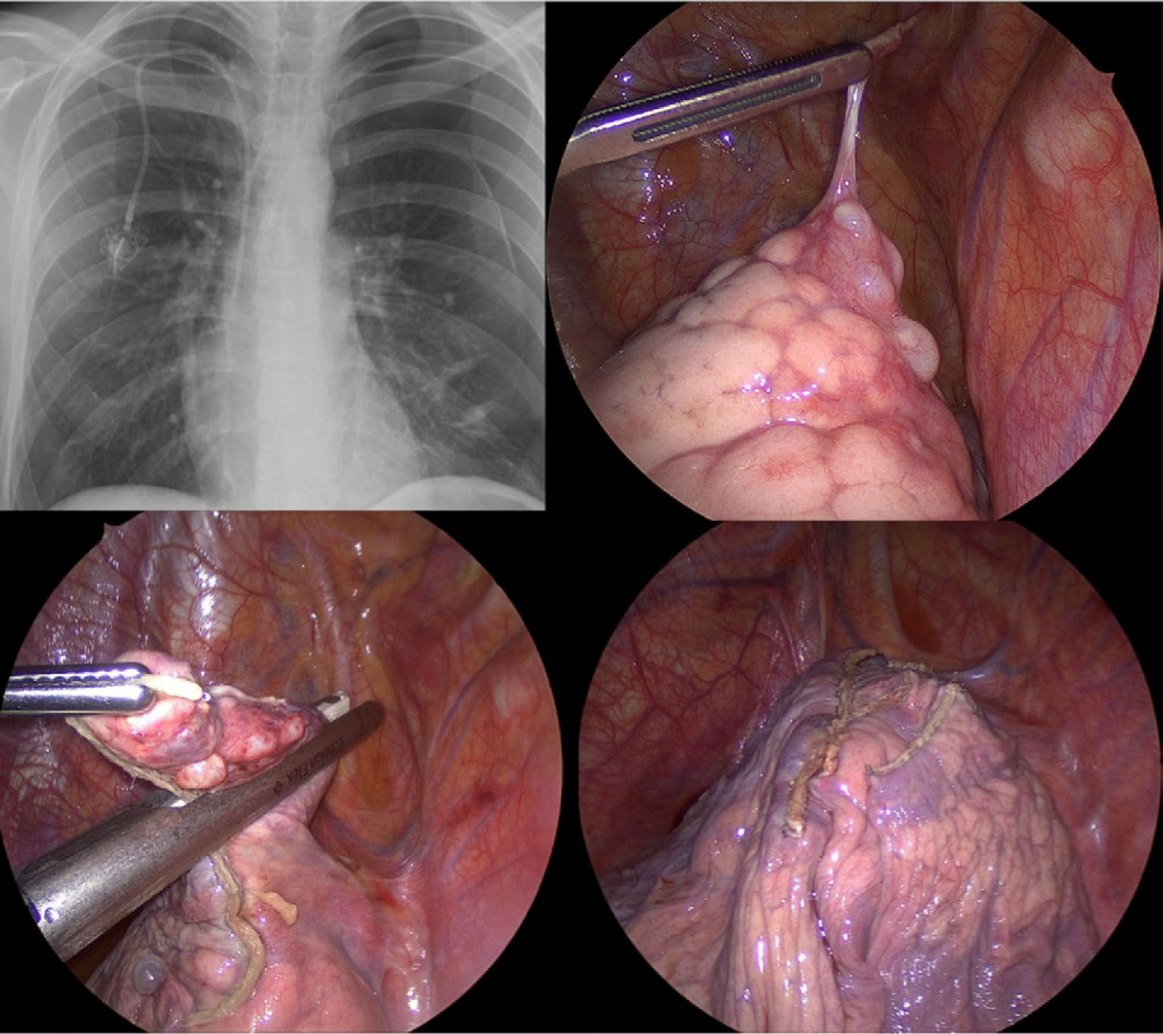
Pre‐operative chest radiograph of a child with acute lymphoblastic leukemia and recurrent pneumothorax. The patient underwent video‐assisted thoracoscopic surgery (VATS) for blebectomy and chest tube placement. This case highlights the role of minimally invasive thoracic approaches in managing pulmonary complications during therapy.

Thoracoscopy is another use of MIS in children with hematologic malignancies. It can be used as a diagnostic or therapeutic tool for biopsies, malignant pleural effusions, or mass resections [62, 63]. Video‐assisted thoracoscopic surgery (VATS) provides a less invasive but effective way to biopsy or resect thoracic masses without an increase in recurrence compared to open thoracotomy [64]. Similarly, VATS decreases morbidity when managing cancer sequelae. Pediatric surgeons often place a camera in the thoracic cavity to manage malignant pleural effusions, either to evacuate fluid or for assistance in the proper positioning of a chest tube. If pleural effusion is the presenting symptom, VATS is also an effective diagnostic tool, with a diagnostic yield of 90% via cytology or targeted pleural biopsies [65, 66]. Additionally, pleurodesis after recurrent pneumothorax (Figure 2) can easily and efficiently be performed via VATS [67]. In children who are neutropenic and immunosuppressed, using minimally invasive techniques can provide a less morbid treatment option and theoretically maintain oncologic timelines while minimizing complications.

## 6 Indocyanine Green Utilization in Lymph Node Biopsy

**FIGURE 3:**
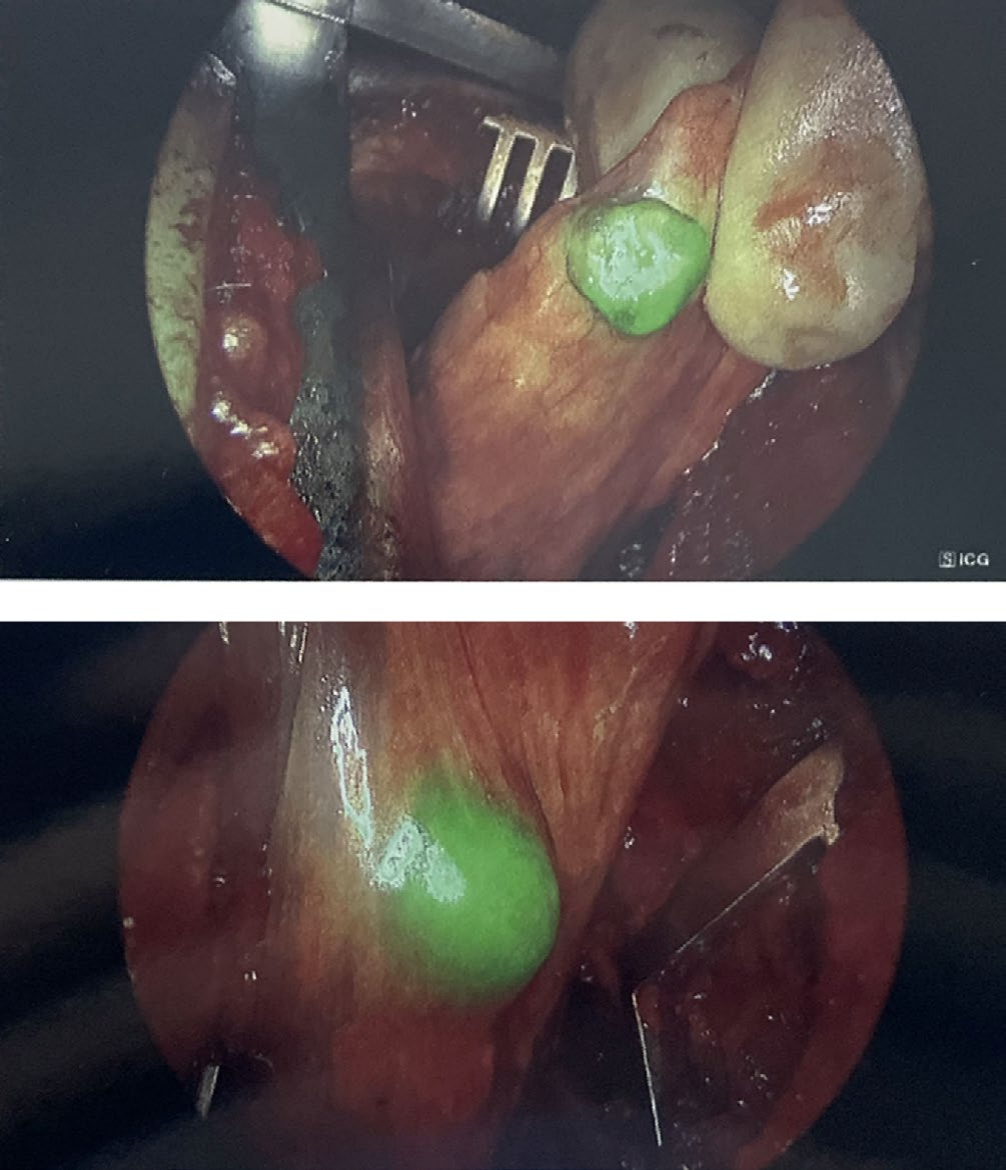
Intraoperative findings in a child with a large intraabdominal Burkitt's lymphoma and associated pleural effusion. The patient underwent video‐assisted thoracoscopic surgery (VATS) for pleural fluid drainage and chest tube placement, followed by diagnostic laparoscopy with tissue biopsy and peritoneal fluid sampling. This case demonstrates the combined use of minimally invasive approaches to reduce morbidity and expedite return to chemotherapy.

The utilization of indocyanine green (ICG) (Figure 3) in operative settings serves as a tool for real‐time visualization and identification of anatomical features [68]. One particular role for ICG in hematologic malignancies is for lymph node identification in excisional biopsies. Pediatric surgeons are often called to assist with obtaining tissue for suspected lymphomas, which can be challenging depending on the location and size of the primary tumor. ICG demonstrates fluorescence in sentinel lymph node biopsies across multiple solid organ tumors (pediatric melanoma, sarcoma, and squamous cell carcinoma) with detection rates of 95%, indicating its utility in identifying nodes with accuracy and sensitivity [69, 70]. In addition to sentinel lymph nodes, ICG has been utilized for retroperitoneal node identification. In four patients with paratesticular rhabdomyosarcoma, a minimally invasive retroperitoneal lymphadenectomy was performed under ICG guidance [71]. ICG injection allowed for intraoperative lymph node mapping and identification, facilitating a minimally invasive approach to a difficult retroperitoneal dissection and allowing for same‐day discharge of all four patients. In patients with intraabdominal lymph nodes requiring excision for tissue diagnosis, ICG can allow for visualization of nodes and can facilitate a minimally invasive surgical approach with laparoscopy.

## 7 Intraoperative Navigation

Advances in intraoperative navigation are now more prevalent, as more pediatric surgeons are adopting minimally invasive techniques. Emerging technologies such as augmented reality (AR) and image‐guided navigation aim to provide a more accurate method for biopsies and tumor resection. This technology is particularly important in the mediastinum, where hematologic malignancies can lie near vital structures. Intraoperative navigation integrates preoperative imaging (CT or MRI) with intraoperative visualization to demarcate the margins of tumor versus normal tissue. Visibility in these cases is often poor due to the constrained space and difficulty discerning tumor tissue planes. The margin for error is extremely small and makes precise surgical maneuvering a crucial part of mediastinal tumor resections. A small case series has found that AR can be safely used for complete resection of mediastinal masses even in patients who underwent chemotherapy in the past, which would normally cause significant tissue distortion [72, 73].

## 8 Robotic Surgery

While the majority of this review has focused on laparoscopic and thoracoscopic surgery, an important emerging technology in MIS is the utilization of robotics as an adjunct. Robot‐assisted surgery is becoming increasingly utilized in pediatric patients across a variety of clinical problems, including general surgery (hernia repairs, cholecystectomies) and surgical oncology (tumor resections, lymphadenectomies) [74]. For patients with hematologic malignancies, one essential consideration for robotics is lymph node biopsy. As discussed previously, the use of ICG to identify lymph node targets is a helpful adjunct for lymph node biopsies or lymphadenectomies. The robotic platform has a built‐in modality for ICG fluorescence, allowing easy utilization during resections. Additionally, robotic surgery has been demonstrated to be a safe and effective modality for splenectomy and should be considered. A single‐study retrospective review noted that robotic‐assisted splenectomy resulted in a decreased length of stay compared to even laparoscopic splenectomy [75]. Similarly, a systematic review of robotic‐assisted splenectomy demonstrated no differences in conversion rate, mean operative time, or rate of complications compared to laparoscopic approaches [76]. Robotic‐assisted surgery is an emerging technology across pediatric surgery and should be considered as an approach for patients with hematologic malignancies for diagnosis and treatment of a variety of conditions.

## 9 Pearls and Pitfalls

When feasible, MIS at diagnosis or during treatment can enable earlier initiation of therapy and a faster return to treatment because recovery is typically quicker. It has been associated with less postoperative pain, shorter hospital stays, and lower morbidity than open procedures across many pediatric surgical indications. In hemodynamically stable patients, MIS can be considered before laparotomy to evaluate the abdominal cavity and refine the operative plan.

MIS, however, is not appropriate in all cases. Hemodynamically unstable patients—such as those with frank perforation—are better served by laparotomy, which provides faster access, improved visualization, and simpler management of complex injuries. Laparoscopy may also be unsuitable for complex typhlitis or large Burkitt lymphoma masses because of size or invasion of adjacent structures, and large mediastinal masses are often difficult to resect via VATS.

**FIGURE 4:**
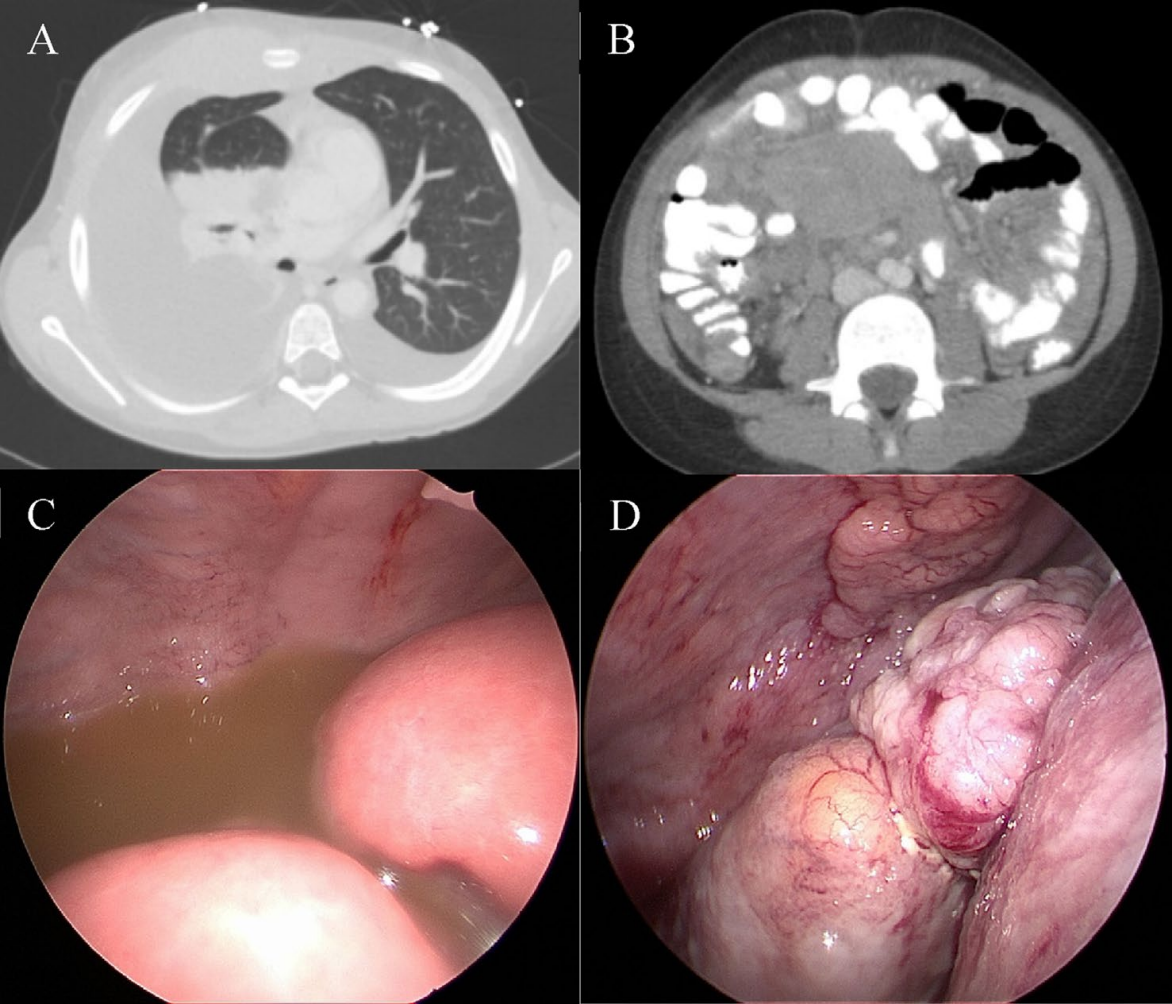
Pre‐operative and intraoperative imaging of a child with Burkitt's lymphoma and pleural effusion. (A) Pre‐operative chest radiograph demonstrating a large pleural effusion. (B) Abdominal CT scan showing intraabdominal Burkitt's lymphoma. (C) Diagnostic laparoscopy with peritoneal cytology. (D) Laparoscopic‐guided tissue biopsy of the mass. Together, these images illustrate the complementary role of VATS and laparoscopy in managing thoracic and abdominal disease while minimizing surgical morbidity.

Some scenarios require multiple minimally invasive techniques, which can reduce physiologic stress. For example (Figure 4), a child with a large intra‐abdominal Burkitt's lymphoma and a pleural effusion underwent VATS for fluid drainage and chest‐tube placement, followed by diagnostic laparoscopy with tissue biopsy and peritoneal fluid drainage. This case illustrates how MIS can minimize morbidity and support a rapid return to therapy. Overall, MIS should be considered in hematologic malignancies for diagnostic biopsies, management of common surgical problems, and—when appropriate—definitive treatment.

## 10 Conclusion

While hematologic malignancies are primarily treated with medical therapies, a role remains for minimally invasive interventions for these patients. MIS approaches can be utilized for diagnosis via lymph node biopsies, for the management of common surgical problems, and for the initial intervention of intraabdominal complications. The utilization of MIS in pediatric patients with hematologic malignancies is a safe, appropriate, and helpful approach for a variety of surgical problems. Limitations of the present evidence include the absence of prospective, large‐scale studies, underscoring the need for multicenter collaboration to confirm safety and efficacy and to rigorously evaluate future directions such as AR and robotic surgery.

## Author Contributions

All authors contributed to the conception and design of the review. J.C.F. drafted the initial manuscript. A.C., K.G.M., and H.N.L. contributed to the literature review, critical revisions, and intellectual content. All authors reviewed, edited, and approved the final version of the manuscript.

## Conflicts of Interest

The authors declare no conflicts of interest.

## Data Availability

The article is a narrative review and does not report any new data. No datasets were generated or analyzed during the current study.
